# A universal, high‐performance ECG signal processing engine to reduce clinical burden

**DOI:** 10.1111/anec.12993

**Published:** 2022-07-29

**Authors:** Austin Gibbs, Matthew Fitzpatrick, Mark Lilburn, Holly Easlea, Jonathan Francey, Rebecca Funston, Jordan Diven, Stacey Murray, Oliver G. J. Mitchell, Adrian Condon, Andrew R. J. Mitchell, Benjamin Sanchez, David Steinhaus

**Affiliations:** ^1^ The Allan Lab Jersey General Hospital Saint Helier Jersey; ^2^ B‐Secur Ltd Belfast UK; ^3^ Department of Electrical & Computer Engineering University of Utah Salt Lake City Utah USA; ^4^ Kansas City Missouri USA

**Keywords:** artifact removal, ECG, noise filtering, QRS detection, signal conditioning, signal processing

## Abstract

**Background:**

Electrocardiogram (ECG) signal conditioning is a vital step in the ECG signal processing chain that ensures effective noise removal and accurate feature extraction.

**Objective:**

This study evaluates the performance of the FDA 510 (k) cleared HeartKey Signal Conditioning and QRS peak detection algorithms on a range of annotated public and proprietary ECG databases (HeartKey is a UK Registered Trademark of B‐Secur Ltd).

**Methods:**

Seven hundred fifty‐one raw ECG files from a broad range of use cases were individually passed through the HeartKey signal processing engine. The algorithms include several advanced filtering steps to enable significant noise removal and accurate identification of the QRS complex. QRS detection statistics were generated against the annotated ECG files.

**Results:**

HeartKey displayed robust performance across 14 ECG databases (seven public, seven proprietary), covering a range of healthy and unhealthy patient data, wet and dry electrode types, various lead configurations, hardware sources, and stationary/ambulatory recordings from clinical and non‐clinical settings. Over the NSR, MIT‐BIH, AHA, and MIT‐AF public databases, average QRS Se and PPV values of 98.90% and 99.08% were achieved. Adaptable performance (Se 93.26%, PPV 90.53%) was similarly observed on the challenging NST database. Crucially, HeartKey's performance effectively translated to the dry electrode space, with an average QRS Se of 99.22% and PPV of 99.00% observed over eight dry electrode databases representing various use cases, including two challenging motion‐based collection protocols.

**Conclusion:**

HeartKey demonstrated robust signal conditioning and QRS detection performance across the broad range of tested ECG signals. It should be emphasized that in no way have the algorithms been altered or trained to optimize performance on a given database, meaning that HeartKey is potentially a universal solution capable of maintaining a high level of performance across a broad range of clinical and everyday use cases.

## Graphical abstract

A universal ECG signal processing engine with highly effective noise removal and accurate QRS detection capability enables the acquisition of medical‐grade ECG data on a wide range of ECG‐functionalized devices.

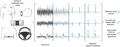

## 1 INTRODUCTION

Cardiovascular Disease (CVD), an umbrella term encompassing an array of disorders affecting the heart and blood vessels, is the leading cause of death worldwide and a significant burden on global health care.(World Health Organisation (WHO), 2021) Early detection and monitoring of CVDs is crucial as it allows the identified conditions to be treated and appropriate medical precautions to be established. Due to the wealth of physiological information derived from the heart's electrical signal, electrocardiography is among the most effective diagnostic tools available to aid clinicians in the fight against CVD. Although once restricted to clinical settings, integrating ECG functionality into portable devices allows healthcare professionals to continuously monitor cardiac function remotely over extended periods. The ambulatory approach is compelling and is becoming increasingly valuable in diagnosing and managing cardiac arrhythmias, including atrial fibrillation (AF), which manifest infrequently and inconsistently. (Sana et al., 2020) Being able to accurately extract the relevant physiological information from patients amidst the background noise of a non‐clinical, unstable environment is vital to ensure no further increases in burden to the clinical pathway.

Electrocardiogram (ECG) signals are characterized by five key features (P, Q, R, S and T waves) pertaining to the direction of electrical signal propagation through the heart at various stages of the cardiac cycle. Variations in these characteristic waveforms' morphology, orientation and frequency can indicate various cardiac conditions, such as arrhythmias and ischemic heart disease, among others. Computer‐aided ECG algorithms that process, interpret and autonomously diagnose cardiac abnormalities have emerged as powerful tools to support manual diagnosis by specialists. The QRS complex, which represents ventricular depolarization, is the most prominent waveform in the ECG and the easiest for algorithms to detect due to its high amplitude.(do Vale Madeiro et al., 2019) Accurate and reliable algorithmic detection of the QRS complex is crucial as it serves as the basis from which: (a) other characteristic waveforms (P & T waves) in the ECG can be identified, and (b) critical diagnostic parameters can be derived. The efficient and accurate extraction of the latter is essential as such information acts is the foundation from which more complex algorithms can be constructed. For instance, beat to beat (R‐R) intervals are obtained by measuring the time between correctly detected QRS signals and can be used to calculate heart rate (HR), heart rate variability (HRV) and act as an input for arrhythmia detection algorithms.

Although clinical recording protocols are standardized, QRS signal morphology can vary significantly from patient to patient.(Corrado et al., 2009; Rijnbeek et al., 2014) To maximize compatibility with QRS detection algorithms, minimizing noise contamination on the ECG signal is essential as it allows the QRS complex to be readily distinguished. Noise contamination can arise from various sources, including 50/60 Hz power line interference, the electrode‐skin interface, muscle activity, and general motion artifact noise induced by patient movement. In the context of automated ECG detection algorithms, noise artifacts are especially problematic as they can trigger false‐positive events that obscure valid ECG metrics.

As automated detection algorithms become more common, there is a clear need to input high‐quality data to ensure they function to a high‐performance level. This need is exacerbated in ambulatory monitoring applications, as the levels of noise artifacts produced during daily activities are significantly greater than in a hospital setting.(Kumar et al., 2018) Effective signal conditioning algorithms must be carefully designed to ensure that; (a) noise artifacts are not falsely classified as QRS complexes, and (b) true QRS complexes are not removed alongside noise during filtering steps. Noise artifacts can obstruct the distinction of true QRS complexes, potentially leading to the missed detection of an important pathological event that can delay or prevent the diagnosis of a cardiac abnormality. Therefore, an effective signal conditioning step must follow the acquisition of raw ECG data to remove excess noise and output ECG signals from which the QRS can be correctly identified, and critical diagnostic parameters obtained.

Despite the plethora of QRS detection algorithms introduced over the past few decades, there still lacks a universal algorithm capable of operating with high accuracy across the wide range of clinically relevant use cases. In this study, we introduce the HeartKey® Signal Conditioning and QRS Detection algorithms and evaluate their performance on a total of 14 ECG databases, chosen to represent the inevitable variability in signal quality of real‐world ECG data. Across the broad range of use cases, 3,135,366 annotated beats were analyzed in total. HeartKey demonstrated highly adaptable QRS detection accuracy and positive predictivity in all cases. Due to the low memory footprint and processing requirements of the algorithm, it has the potential to be employed in a host of ECG monitoring applications, both inside and outside of a clinical environment.

## 2 METHODS

### 2.1 HeartKey algorithm overview

**FIGURE 1:**
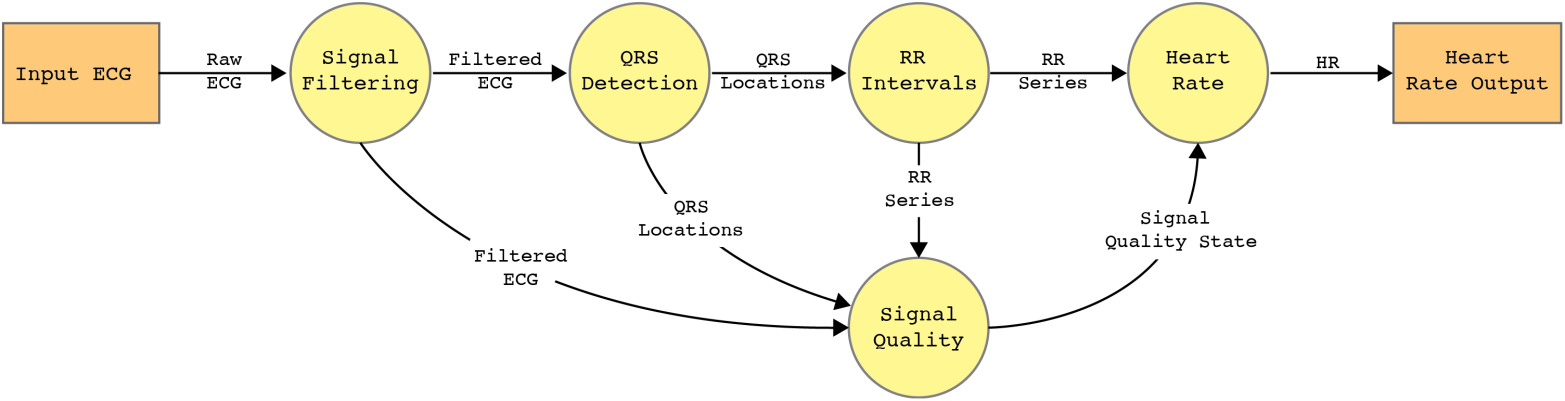
Flow of ECG data through HeartKey algorithm(s)

HeartKey QRS Detection and Heart Rate algorithms are FDA 510 (k) cleared as a Class II medical device. Operationally, the algorithms consist of several distinct stages, detailed in Figure 1, designed to produce reliable and robust performance from raw ECG data across a range of ECG lead configurations and hardware sources. The input signal is initially processed through a signal filtering step that has been fine‐tuned to operate on signals from various electrode materials and hardware sources. This ensures that the variation in signal quality associated with both methods is adequately dealt with and that the information to be extracted from the processed data is accurate, reliable and, therefore, valuable at a clinical level. After filtering, a clean signal is fed through the QRS detection algorithm. Upon successful identification of the QRS locations, an R‐R interval series can be calculated. This feeds into, among others, the HR algorithm. Throughout the process, the HeartKey algorithms employ a variety of methods to assess each calculated metric for validity; this ensures robustness and accuracy on even the noisiest of signals. The HeartKey algorithms will also assess and provide an indicator of the ECG signal quality, only outputting a HR value if the signal is deemed to be of sufficient quality.

### 2.2 wet electrode ECG data overview

Five public PhysioNet wet electrode databases were chosen to evaluate the performance of HeartKey QRS Detection and Signal Conditioning algorithms: the NSR database, the MIT‐BIH database, the MIT‐AF database, the AHA database, and the NST database.(Goldberger et al., 2000) The respective databases include 179 raw ECG files, 3,086,647 annotated beats, with a variety of healthy and unhealthy patients and ECG morphologies on both clean and noisy signals. The databases were tested in line with the AAMI/ANSI EC57 standard (CENELEC ‐ EN 60601–2‐27, 2014) – a medical standard that pertains to a “protocol for a reproducible test with clinical requirements and emphasizes the record‐by‐record presentation of results; that reflect an algorithm's ability to detect events of clinical significance.” Proprietary wet electrode data were also collected on an industry gold standard ambulatory device (Bittium Faros 180)(Bittium, 2018) using a wet electrode lead II configuration.

### 2.3 Dry electrode ECG data overview

To demonstrate the applicability of the HeartKey Signal Conditioning and QRS Detection algorithms for integration into the ever‐increasing range of ECG‐functionalized dry electrode hardware, ECG data originating from a variety of devices and challenging collection protocols, including walking, and running, were used. This database represents the real world, where ECGs are performed in various settings, through various methods, by various operators, which ultimately results in a significant variation of signal quality. Challenging signals in the database include those with a significant degree of high frequency noise, motion artifacts, low QRS amplitude, irregular rhythms, and variable beat morphologies. Performance on this dry electrode ECG data was evaluated against manual peak annotations and where appropriate, compared to data collected on an industry gold standard ambulatory wet electrode device (Bittium Faros 180).

### 2.4 ECG data annotation

With the exception of the MIT‐AF database, PhysioNet databases have been independently annotated by cardiologists and the performance of HeartKey was generated against these annotations. Beat annotations for the MIT‐AF DB were generated by a minimum of two separate annotators using a computer‐based annotation tool. This was followed by a group review of any outstanding annotations, during which highlighted discrepancies were resolved. Proprietary dry electrode databases were manually annotated by board‐certified cardiologists. Databases were annotated individually, followed by a similar group review to ensure agreement on annotations for challenging signals. As manual annotation is the gold standard for ECG performance analysis, these annotations were used as a criterion by which the HeartKey Signal Conditioning algorithm and QRS detection performance was compared.

### 2.5 Data processing

Each ECG file was individually processed through the HeartKey Signal Conditioning algorithm. QRS Detection results were generated using PhysioNet WFDB programs bxb and sumstats. As standard QRS accuracy measurements employ a wide error window (+/− 150 ms), the precise location of detection within the QRS complex is not important, only that this location remains consistent from beat to beat. Looking at this in isolation could mask variation in where the algorithm picks up the beat. It is, therefore, beneficial to include HR accuracy measurements. Many measurements exist for HR, and none are universally accepted. As recommended in Section 4.3.3.1 of ANSI EC57, HR statistic reference annotation files were created for each record, calculating the HR from the reference beat annotations with the same method used in the device. The comparison will generate the Root Mean Square Heart Rate Error to measure the error between the reference and test annotations. Heart Rate Error statistics are generated using WFDB programs mxm and sumstats.

### 2.6 Performance metrics

There are four outcomes in which the detector is presented with an input that is either an event or a non‐event:

- True positive (TP) is an event detected correctly*.
- False‐negative (FN) is a missed event*.
- False‐positive (FP) is a non‐event detected as an event.
- True negative (TN) is a non‐event correctly rejected.

*A correctly detected event is defined as a QRS detection location within 150 ms of the QRS annotation, as stated within ANSI (AAMI EC67‐2012) standards. If QRS detection is outside the 150 ms window, the beat is missed and classified as a false‐negative.

The most common detector performance measures are sensitivity (Se) and positive predictive value (PPV), as detailed below. Sensitivity relates to the ability of the algorithm to identify true events correctly and is calculated using the following equation:

$$\mathrm{Se}\;\left(\%\right)=\left(\frac{\mathrm{TP}}{\mathrm{TP}+\mathrm{FN}}\right)\times 100$$

PPV relates to the algorithm's ability to avoid incorrectly detecting false events and is calculated using the following equation:

$$\mathrm{PPV}\;\left(\%\right)=\left(\frac{\mathrm{TP}}{\mathrm{TP}+\mathrm{FP}}\right)\times 100$$

Root Mean Square HR Error allows comparison of the HeartKey HR information relative to the reference value.

$$\text{RMSE}=\sqrt{\sum_{i=1}^{N}\frac{{\left(\text{Actual}\;\mathrm{HR}-\text{Annotated}\;\mathrm{HR}\right)}^{2}}{N}}$$

## 3 RESULTS AND DISCUSSION

### 3.1 HeartKey performance on wet electrode data

**FIGURE 2:**
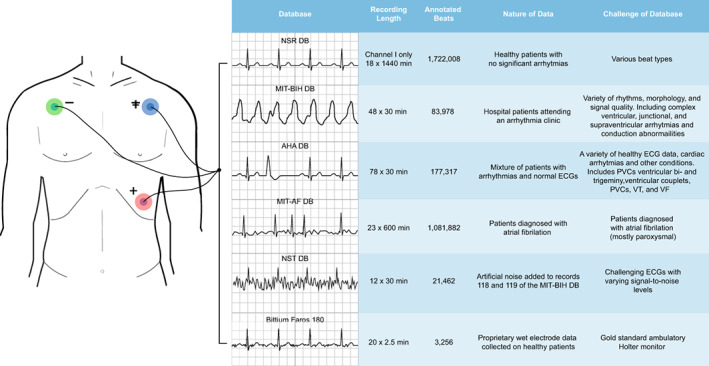
Overview of data from wet electrode databases

Numerous algorithms have reported excellent performance statistics (>99% QRS Se & PPV) on databases for which they have undergone a learning period,(Cai & Hu, 2008; Xiang et al., 2018; Xue et al., 1992) or when designed to give optimal performance on a given database.(Farashi, 2016; Pan & Tompkins, 1985; Rahul et al., 2021) Without cumbersome training periods, relatively few algorithms have been reported to retain a high performance across multiple databases with different morphologies, cardiac conditions, and signal qualities.(Dotsinky & Stoyanov, 2004; Kim & Shin, 2016; Kunzmann et al., 2002) HeartKey Signal Conditioning and QRS detection algorithms were initially evaluated on five public PhysioNet databases: NSR DB, MIT‐BIH DB, AHA DB, MIT‐AF DB and NST DB. The majority of ECG data in these databases was collected using a wet electrode‐modified limb lead II setup. Each database possesses inherent challenges for the HeartKey algorithm to overcome, outlined in Figure 2.

The NSR database contained ECG data with high signal quality and no significant arrhythmias and was included to demonstrate the performance of HeartKey on optimal wet electrode ECG data. HeartKey achieved QRS detection Se and PPV of 99.84% and 99.40%, respectively, on this healthy dataset. MIT‐BIH is a benchmark database of ambulatory ECG recordings containing various arrhythmias and cardiac abnormalities. It is by far the most frequently used database to validate the performance of signal conditioning algorithms in the literature.(Moody & Mark, 2001) For this study, Channel I of each recording was analyzed. The 5‐minute training period at the beginning of each record was excluded from analysis as HeartKey does not require a learning phase. The AHA database is another popular public ECG database that contains a range of rhythms, including NSR, alongside numerous less common arrhythmias. Over the two databases, virtually all significant, clinically relevant arrhythmias are covered, ranging from mild conditions, such as tachycardia, to life threatening heart rhythms like ventricular fibrillation. This gives an excellent indication of HeartKey's performance on the wide array of real‐world clinical ECG data. On both databases, HeartKey achieved >99.60% QRS PPV. QRS Se performance values of 98.96% and 97.43% were, respectively, obtained for MIT‐BIH and AHA databases. When considering the clinical impact 97% QRS Sensitivity would have in the “worst case” scenario (at a maximum HR of 200 bpm), this equates to approximately 2–3 missed or extra beats within a 30s recording. In relation to arrhythmia detection applications, this would not be deemed to be clinically significant.(Bouzid et al., 2022) RMS HR error values of 1.09% and 1.93% were achieved, respectively, for the MIT‐BIH and AHA databases.

Although MIT‐BIH and AHA databases do contain ambulatory ECG records, these datasets are among numerous other recording protocols. This means that the average QRS detection statistics are not truly representative of HeartKey performance on ambulatory data, which can be considerably more challenging due to inherent noise contamination through patient movement. To demonstrate the utility of HeartKey on ambulatory wet electrode ECG data, the MIT‐AF database, which contains long term, continuous ECG data from patients diagnosed with atrial fibrillation (AF), was next analyzed. HeartKey achieved QRS Se of 99.38% and QRS PPV of 97.37% on this ambulatory dataset. To further highlight the high performance of HeartKey on Holter wet electrode data, a proprietary dataset was collected on healthy subjects using a Bittium Faros 180, a gold standard ambulatory recording device. Similar performance statistics were achieved on this dataset with an average QRS Se and PPV of 99.86% and 99.66%.

The ability of HeartKey to successfully detect the QRS complex amidst various levels of noise was evaluated using the Noise Stress Test (NST) database. In this public dataset, artificial noise is overlayed on two clean ECG signals from the MIT‐BIH Database (records 118 and 119), to emulate baseline wander, muscle movement artifacts and electrode motion artifacts. HeartKey achieved a QRS Se of 93.26% and PPV of 90.53% on the NST DB. Understandably, performance is lower than the other four PhysioNet databases detailed in Table 1; however, HeartKey still performed well even with the extreme presence of noise. Individual records from the NST database were analyzed to determine the level of noise at which the performance of the algorithm becomes significantly affected. QRS Se and PPV performance values remain above 99% for signal‐to‐noise ratio (SNR) levels as low as 12 dB. At an SNR of 6 dB, the average Se and PPV reduce to 98.26% and 95.18%, respectively. At 0 dB and − 6 dB, the performance deteriorates markedly when noise becomes equal to or greater than the ECG signal.

**TABLE 1:** Performance of HeartKey on wet electrode databases

Measurement	NSR DB	MIT‐BIH DB	AHA DB	MIT‐AF DB	NST DB	Bittium faros 180
Detected Beats	1,719,427	82,996	173,117	1,075,071	20,000	3521
False Positives	10,134	111	618	28,315	2141	12
False Negatives	2581	982	4200	6811	1462	5
QRS Average Se (%)	99.84	98.96	97.43	99.38	93.26	99.86
QRS Average PPV (%)	99.40	99.86	99.60	97.37	90.53	99.66
RMS HR Error (%)	1.09	1.93	5.42	3.21	19.42	0.61

### 3.2 HeartKey performance on dry electrode data

Wearable devices with ECG functionality are emerging as powerful tools to detect and remotely monitor cardiac abnormalities outside of a clinical environment.(Bouzid et al., 2022) Such devices typically measure the heart's electrical signal using a dry electrode single‐lead ECG setup. However, data collection on dry electrode ECG wearables is inherently more challenging for two reasons: the positioning of the wearable device at peripheral locations on the body, such as the wrist or hands, can lead to a reduction in signal amplitude, whereas the increased impedance given by dry electrodes leads to enhanced noise interference. Prior to an effective signal processing step, the combined issues produce data in which the relevant ECG waveforms are buried under noise. The lack of redundancy in single‐lead ECG signals further stresses the need for accurate and reliable signal processing algorithms to extract the maximum amount of diagnostic information from challenging ECG traces.

**FIGURE 3:**
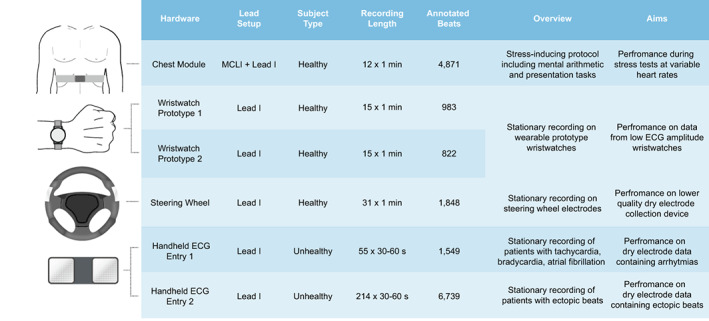
Overview of data from dry electrode databases

To demonstrate the broad applicability of HeartKey signal conditioning and QRS detection algorithms, ECG data was collected on a range of dry electrode devices. The collected datasets contain various challenging single‐lead ECG signals, including those with high frequency noise, motion artifacts, low QRS amplitude, irregular beats, and irregular rhythms to represent real‐world dry electrode data as closely as possible (Figure 3).

HeartKey QRS detection algorithm displayed strong performance on the range of dry electrode use cases, with an average QRS Se and PPV of 99.13% and 99.00% over the six tested databases (Table 2). The highest QRS detection performance was observed with the chest module, with an average QRS Se of 99.95% and PPV of 99.94%. This is unsurprising as the large dry electrode surface area ensures continuous contact with the skin. As the device is securely strapped in place, there is also less electrode‐skin contact movement; and therefore, fewer noise artifacts. The proximity of the device to the heart also allows a high amplitude QRS to be recorded. This hardware is a stark contrast to prototype wristwatches 1 and 2, which feature dry electrodes with smaller surface areas located at a peripheral site on the body (wrist), resulting in a low amplitude ECG that is more prone to noise contamination. Despite these challenges, HeartKey achieved QRS Se (>99.54%) and PPV (>98.29%) on both tested prototype wristwatches. The steering wheel similarly suffers due to the proximal location of data, its data collection (hands/fingers) and as subjects are required to grip the wheel in an unsecured manner, there is inevitable noise contamination from muscle contractions and the moving electrode‐skin interface. Average QRS Se and PPV values of 99.29% and 99.07% were achieved despite these difficulties. The most challenging dry electrode datasets in Table 2 are arguably those collected with the handheld ECG device (Entries 1 & 2). The electrode‐skin contact site for these use cases is at the extremity of the body (fingertips), resulting in a low ECG amplitude. To add further difficulty, the datasets were also collected on unhealthy patients and contain various arrhythmias and ectopic beats. HeartKey achieved QRS Se of 97.98% and 98.14% and QRS PPV of 99.30% and 98.65% on handheld ECG device entries 1 & 2, respectively.

**TABLE 2:** Performance of HeartKey on dry electrode databases

Hardware	Detected beats	False positives	False negatives	QRS average sensitivity (%)	QRS average PPV (%)	RMS HR error (%)
Chest Module	4869	3	2	99.95	99.94	0.63
Wristwatch Prototype 1	978	22	5	99.54	98.29	0.98
Wristwatch Prototype 2	821	13	1	99.87	98.63	1.23
Steering Wheel	1836	16	12	99.29	99.07	1.86
Handheld ECG Entry 1	1522	7	217	97.98	99.30	2.00
Handheld ECG Entry 2	6628	115	111	98.14	98.65	2.57

### 3.3 HeartKey performance on Motion‐Based dry electrode data

Diagnosing intermittent cardiac arrhythmias is a challenge. These arrhythmic episodes can occur infrequently and unpredictably, and they generally require prolonged and repeated cardiac monitoring to be successfully detected.(Heidt et al., 2016) Performing this requires the development of reliable and clinically safe ambulatory monitoring methods. To maximize patient compliance and obtain real‐world ECG data, the chosen hardware device needs to be discreet, lightweight, and unobtrusive so the patient can continue as close to as possible an uninterrupted daily life routine. However, this requirement needs to be balanced against maximizing data quality to ensure that false positives and negatives are eliminated as much as possible.

Wearable dry electrode ECG setups are ideal for discreetness and allow patients to maintain regular daily routines. For the ECG functionality within the wearable device to be of clinical value, the algorithms must be capable of maintaining a high performance across the spectrum of motion‐based scenarios the patient will enact each day—such as walking or exercising—which will invariably alter ECG signal quality. The performance of HeartKey algorithms was investigated on internally collected ECG data recorded on consumer‐grade chest strap electrodes (MCLI) during two motion‐based protocols (Table 3). In the first, subjects were instructed to walk on a treadmill (6 km/h) for 4 min. In the second protocol, after an initial warm up period, subjects were asked to run on a treadmill at increasing speeds (subject dependent) over 4 min. As expected, the raw ECGs are of poor quality, suffer from frequent baseline wander, and contain large amounts of noise artifacts arising from dry electrode‐skin contact movement and patient muscle activity. HeartKey signal processing and QRS detection algorithms performed well on these challenging datasets, achieving a QRS Se and PPV of 99.09% and 98.15% during the walking protocol (Entry 1), and a QRS Se and PPV of 99.86% and 99.87% during the running protocol. Although the performance was expected to be lower during the running protocol due to the increased levels of motion, the opposite was observed. The buildup of sweat at the electrode‐skin interface during the running protocol could explain the results, as this would lead to greater conductance and essentially allow it to act as a wet electrode. HeartKey's ability to improve the quality of dry electrode ECG data without interrupting the patient's daily routine will facilitate clinical‐grade ambulatory monitoring, hugely enhancing the breadth and depth of available diagnostic hardware and improving their performance both in and out of hospital settings.

**TABLE 3:** Performance of HeartKey during motion‐based ECG recording protocols

Hardware	Electrode setup	Protocol overview	Recording length	Annotated beats	QRS average sensitivity (%)	QRS average PPV (%)	RMS HR error (%)
Chest Module Entry 1	Dry MCLI + Lead I	Walking on a treadmill at increasing speeds	21 × 4 min	8858	99.09	98.15	1.56
Chest Module Entry 2	Dry MCLI + Lead I	Running on a treadmill increasing speeds	189 × 1 min	19,793	99.86	99.87	0.48

## 4 CONCLUSION

The ability of ECG signal conditioning algorithms to achieve relatively high performance on wet electrode databases for which they have been optimized or trained is not uncommon. However, most ECG signal conditioning algorithms lack universality and are incapable of maintaining the same high level of performance over multiple databases and use cases, limiting their application. We have shown that HeartKey is a crucial tool in pursuing a universal approach to ECG signal conditioning, showing accurate and reliable QRS detection performance across a broad range of clinically relevant datasets. It should again be emphasized that HeartKey algorithms required no learning phases, and in no way have been adjusted to perform better on the tested databases. As with many clinical ECGs, the data are there, but the signal can be of poor quality and hidden under a range of noise. Prior to the development of high‐quality signal conditioning, these data were lost or not actionable. Patients and clinicians could go through multiple repeated investigations, extended periods of monitoring or, in some cases, the condition could be overlooked until a more catastrophic cardiac event occurs. Effective signal conditioning with HeartKey allows clinicians to extract the right data, making the crucial intervention without repeated investigations.

## 5 AUTHOR CONTRIBUTION

**Austin Gibbs** contributed to investigation, resources, and writing—review and editing. **Matthew Fitzpatrick** contributed to writing—original draft, writing—review and editing, and visualization. **Mark Lilburn** contributed to the validation, methodology, and formal analysis. **Holly Easlea** contributed to the project administration and writing—review and editing. **Jonathan Francey** contributed to the software, methodology, and writing—review and editing. **Rebecca Funston** contributed to the supervision and writing—review and editing. **Jordan Diven** contributed to the validation, methodology, and writing—review and editing. **Stacey Murray** contributed to writing—review and editing, and project administration. **Oliver G.J. Mitchell** involved in data curation. **Adrian Condon** supervised the study. **Andrew R. J. Mitchell**, **Benjamin Sanchez**, **David Steinhau**, and **David Steinhaus** involved in writing—review and editing.

## CONFLICT OF INTEREST

Dr. Austin Gibbs is Lab Director, The Allan Lab, Jersey General Hospital, and is a Consultant to B‐Secur, Ltd (e‐mail: austin@theallanlab.com). Matthew Fitzpatrick (corresponding author) is Scientific Writer, B‐Secur, Ltd (e‐mail: matthew.fitzpatrick@b-secur.com, postal address: B‐Secur. Belfast Ltd, Catalyst Science Park, Belfast, Northern Ireland). Mark Lilburn is Technical Project Manager, B‐Secur, Ltd (e‐mail: mark.lilburn@b-secur.com). Holly Easlea is Data Research Manager, B‐Secur, Ltd (e‐mail: holly.easlea@b-secur.com). Jonathan Francey is Head of Algorithm Development, B‐Secur, Ltd (e‐mail: jonathan.francey@b-secur.com). Jordan Diven is Validation Team Leader, B‐Secur, Ltd (e‐mail: jordan.diven@b-secur.com). Jordan Diven is Validation Team Leader, B‐Secur, Ltd (e‐mail: jordan.diven@b-secur.com). Stacey Murray is Clinical Research Engineer, B‐Secur, Ltd (e‐mail: stacey.murray@b-secur.com). Oliver GJ Mitchell is a Medical Student, The Allan Lab, Jersey General Hospital (e‐mail: mitchogj@gmail.com). Adrian Condon serves as Chief Technology Officer at B‐ Secur, Ltd (e‐mail: acondon@b-secur.com). Dr. Andrew RJ Mitchell is Consultant Cardiologist & Head of Research, The Allan Lab, Jersey General Hospital and is a Consultant to B‐Secur, Ltd (e‐mail: andy@theallanlab.com). Dr. Benjamin Sanchez is Assistant Professor of Electrical and Computer Engineering, University of Utah (e‐mail: benjamin.sanchez@utah.edu). Dr David Steinhaus is a Cardiac Electrophysiologist and Consultant (e‐mail: dmsteinhaus@gmail.com). Dr. Austin Gibbs, Dr. Benjamin Sanchez, Dr. Andrew RJ. Mitchell and Dr. David Steinhaus serve as Scientific Advisors to The Board of B‐Secur, Ltd.

## AUTHOR CONTRIBUTION

**Austin Gibbs** contributed to investigation, resources, and writing—review and editing. **Matthew Fitzpatrick** contributed to writing—original draft, writing—review and editing, and visualization. **Mark Lilburn** contributed to the validation, methodology, and formal analysis. **Holly Easlea** contributed to the project administration and writing—review and editing. **Jonathan Francey** contributed to the software, methodology, and writing—review and editing. **Rebecca Funston** contributed to the supervision and writing—review and editing. **Jordan Diven** contributed to the validation, methodology, and writing—review and editing. **Stacey Murray** contributed to writing—review and editing, and project administration. **Oliver G.J. Mitchell** involved in data curation. **Adrian Condon** supervised the study. **Andrew R. J. Mitchell**, **Benjamin Sanchez**, **David Steinhau**, and **David Steinhaus** involved in writing—review and editing.

## ETHICAL APPROVAL

This study was conducted in accordance with the Declaration of Helsinki. Proprietary data were collected at B‐Secur HQ (Queen's Road, Belfast). Enrolled subjects provided informed consent prior to study participation.

## Data Availability

In‐depth performance statistics from tested databases is available on request from the authors.
